# Protein–ligand binding affinity prediction exploiting sequence constituent homology

**DOI:** 10.1093/bioinformatics/btad502

**Published:** 2023-08-12

**Authors:** Abbi Abdel-Rehim, Oghenejokpeme Orhobor, Lou Hang, Hao Ni, Ross D King

**Affiliations:** Department of Chemical Engineering and Biotechnology, University of Cambridge, Cambridge CB3 0AS, United Kingdom; The National Institute of Agricultural Botany, Cambridge CB3 0LE, United Kingdom; Department of Mathematics, University College London, London WC1H 0AY, United Kingdom; Department of Mathematics, University College London, London WC1H 0AY, United Kingdom; The Alan Turing Institute, London NW1 2DB, United Kingdom; Department of Chemical Engineering and Biotechnology, University of Cambridge, Cambridge CB3 0AS, United Kingdom; The Alan Turing Institute, London NW1 2DB, United Kingdom; Department of Biology and Biological Engineering, Chalmers University of Technology, Gothenburg 412 96, Sweden; Department of Computer Science and Engineering, Chalmers University of Technology, Gothenburg 412 96, Sweden

## Abstract

**Motivation:**

Molecular docking is a commonly used approach for estimating binding conformations and their resultant binding affinities. Machine learning has been successfully deployed to enhance such affinity estimations. Many methods of varying complexity have been developed making use of some or all the spatial and categorical information available in these structures. The evaluation of such methods has mainly been carried out using datasets from PDBbind. Particularly the Comparative Assessment of Scoring Functions (CASF) 2007, 2013, and 2016 datasets with dedicated test sets. This work demonstrates that only a small number of simple descriptors is necessary to efficiently estimate binding affinity for these complexes without the need to know the exact binding conformation of a ligand.

**Results:**

The developed approach of using a small number of ligand and protein descriptors in conjunction with gradient boosting trees demonstrates high performance on the CASF datasets. This includes the commonly used benchmark CASF2016 where it appears to perform better than any other approach. This methodology is also useful for datasets where the spatial relationship between the ligand and protein is unknown as demonstrated using a large ChEMBL-derived dataset.

**Availability and implementation:**

Code and data uploaded to https://github.com/abbiAR/PLBAffinity.

## 1 Introduction

The early stages of drug development rely on finding promising lead compounds. Computational approaches are increasingly utilized to identify such compounds, and molecular docking is a widely used tool for this purpose. Molecular docking deploys different algorithms to generate potential ligand binding poses and resultant affinity estimations within a target binding site (Kroemer 2007). These affinity estimations can be improved using machine learning. A range of scoring methods have been published on the use of machine learning for predicting the resultant binding affinity from a protein–ligand complex (Liu and Wang 2015, Li et al. 2020). These methods are dependent on knowing the exact ligand binding conformation in the binding site since they rely on encoding the spatial relationships between protein and ligand atoms. Alternative methods for calculating protein–ligand binding affinity without spatial information does exist, but to the best of our knowledge, have not been applied to the Comparative Assessment of Scoring Functions (CASF) benchmark datasets (Öztürk et al. 2018, Gao *et al.* 2018, Karimi *et al.* 2019, Tsubaki *et al.* 2019).

Previous studies have made use of the spatial relationship between the ligand and protein constituents in their lowest energy conformation (Jiménez *et al.* 2018, Nguyen and Wei 2019a,b). Currently, one of the latest and most successful descriptors is the extended connectivity interaction fingerprint (ECIF) (Sánchez-Cruz *et al.* 2021). The idea behind this fingerprint is to count pairs of interacting protein and ligand atom types, defined by their specific state and environment. However, ECIFs does not discriminate between close- and long-range contacts, or indeed includes any additional distance information except the tally of all contact pairs within a set distance threshold. Paired distance ECIF (PDECIF) sought to improve upon ECIF by demonstrating that discriminating between close- and long-range interactions would improve predictions. The improvements were indeed significant but not as substantial as one may have expected (Orhobor *et al.* 2022).

These results led us to ask what we could achieve using nonspatial descriptors. Previous studies have highlighted the benefits of adding basic ligand descriptors when predicting binding affinity from a protein–ligand complex (Boyles *et al.* 2020, Sánchez-Cruz *et al.* 2021). Such features are often associated with quantitative structure–activity relationship, a well-established machine learning based methodology where features related to structural, chemical, and physical aspects of ligands are exploited to predict compound biological activity towards a target (Hansch and Fujita 1964, Muratov *et al.* 2020). In this work, we demonstrate that only a small subset of such ligand related features along with basic protein sequence information is required to achieve state of the art results for binding affinity prediction on the CASF datasets.

## 2 Materials and methods

CASF 2007, 2013, 2016, and 2019 datasets were acquired from PDBbind (Cheng *et al.* 2009, Li *et al.* 2014, Su *et al.* 2019). All datasets have a subset of structures included in a ‘refined set’ based on quality-related thresholds. For 2007, 2013, and 2016 versions, the refined datasets have dedicated core sets which conventionally function as test sets, the training sets consists of the remaining structures in the refined datasets. The refined sets for 2007, 2013, 2016, and 2019 contain 1300, 2959, 4057, and 4852 protein–ligand complexes, respectively. The core sets for 2007, 2013, and 2016 contains 210, 195, and 285 complexes, respectively. As the 2019 version lacks a core set, we applied 5-fold cross validation (CV) to test the performance of our descriptors on this dataset.

For each protein structure, a count of the amino acids featured in its active site was performed. The authors of the CASF datasets provide pdb files for all their binding sites, these files include amino acids within 10 Å of the bound ligands. For every protein, each amino acid type was encoded into a vector with the number of occurrences as its corresponding value. Only standard amino acids were considered generating a 20-unit amino acid vector. In addition, eighteen ligand properties were calculated using RDKit (https://www.rdkit.org), these features are listed in Supplementary Table S1. A separate vector containing these calculated values was constructed. Finally, the amino acid and ligand property vectors were concatenated and used as the input data for our machine learning experiments. The target values used were the –log(*Kd*/*Ki*) activity values provided in the index files that accompany the CASF datasets.

At the outset, many CASF ligands were incompatible with RDKit’s sanitation protocol which is used when converting ligands into workable Mol objects. Hence, we first parsed them through PubChem’s standardization service (using PUG REST) (Hähnke *et al.* 2018). This resulted in a nearly complete compatibility with RDKit. The few structures considered invalid by the PubChem standardization process were converted into sybyl mol format using Open Babel (O'Boyle *et al.* 2011). Supplementary materials contain lists of structures incompatible with pubchem’s standardization service for each dataset (Supplementary Table S2).

We performed our experiments using the machine learning method extreme gradient boosted trees in R (https://www.r-project.org). The hyperparameters were optimized using a grid search, and kept all but the number of rounds, max depth, and learning rate as default values, where number of rounds = (500, 1000, 1500, 2000), max depth = (2, 4, 6, 8), and learning rate = (0.001, 0.01, 0.1, 0.2, 0.3). We used internal 5-fold CV on only the training sets to identify the best hyperparameter combination. We report the mean Pearson’s correlation coefficient (*R*), along with the root mean squared error (RMSE) from 5-fold CV. It is worth noting here that this 5-fold CV is different from the internal CV used on the training sets to determine the best hyperparameters.

ChEMBL experiments: amino acid counts were performed for all protein pockets and chains in CASF v.2019 as described above. If a protein had more than one structure present in the dataset, the mean count for these entries was used. Proteins from CASF v.2019 were cross referenced with the ChEMBL database (v.24, 01 May 2018). Ligand properties for RDKit-compatible small molecules (<700 kDa) were calculated using RDKit. Where more than two entries for the same protein–ligand pair were present, only the last entry was kept. −log(*Ki*/*Kd*) was set as the target value and hence, only samples with an associated *Ki*/*Kd* value was included. The resultant dataset consisted of 63 281 compatible ligands across 325 unique proteins. Machine learning was applied as described for the previous experiments. Predictions were performed using the ligand properties, the amino acid counts as well as the two features combined. As the number of drugs per protein is heavily skewed towards a few targets, different thresholds on maximum number of ligands for including targets in the datasets were applied (250, 500, and no limit). Datasets and results are available in a GitHub repository (https://github.com/abbiAR/PLBAffinity).

## 3 Results

Across all datasets, ligand descriptors alone resulted in similar performance, *R* ∼0.68–0.71. Amino acid descriptors performed exceptionally well with the CASF 2007 dataset (Table 1). For the remaining datasets the performance of these amino acid descriptors alone is closer to that seen with ligand properties. The combination of the two descriptors improves the results to varying degrees. For CASF 2007, where amino acid vectors already perform very well, the addition of ligand descriptors only provides a comparatively modest improvement. For CASF 2013, the effect of the combination is more pronounced resulting in a significant improvement, *R* = 0.777, RMSE = 1.480 (Table 1).

**Table 1.** Mean predictive performance of 18 ligand and 20 protein descriptors on the CASF datasets.

Benchmark	RDKit	Amino count	RDKit+amino
CASF-2007	0.713/1.730	0.775/1.583	0.832/1.365
CASF-2013	0.675/1.700	0.646/1.722	0.777/1.480
CASF-2016	0.715/1.551	0.706/1.543	0.844/1.233

a The descriptors are shown separately and in combination (Pearson R/RMSE).

A significant improvement in performance for the CASF 2016 dataset was also seen when the two descriptors are combined, the performance of 0.844 (RMSE = 1.233) outperforms all but one of the state-of-the-art approaches (compare with ECIF and AGL score’s *R* = 0.841 and 0.833, respectively, with the same training data) (Nguyen and Wei 2019a, Sánchez-Cruz *et al.* 2021), it also outperforms the well-known method KDEEP which stands at *R* = 0.82 (Jiménez *et al.* 2018) (see Table 2). PDECIF shows a similar performance with *R* = 0.844 and an RMSE of 1.246 (Orhobor *et al.* 2022). However, this method does result in a slightly lower RMSE (cf. 1.233 and 1.246) and given the set of 38 simple features our method appears highly efficient.

**Table 2.** Comparison with other scoring methods on the CASF 2016 dataset.

Method	Pearson R	RMSE
This work	0.844	1.233
PDECIF	0.844	1.246
ECIF	0.841	1.252
AGL-score	0.833	1.271
KDEEP	0.82	1.27
PLEC-nn	0.817	1.258
ΔVINA-RF20	0.816	1.26
ΔVINAXGB	0.796	1.32
Bappl+	0.71	1.57

a ECIF and PDECIF make use of additional ligand properties in their predictions.

Five-fold CV on the CASF 2019 dataset, which does not have a dedicated test set, reveals that the ligand properties and amino acid counts perform consistently well (Table 3). Again, the combination of these feature vectors results in a significant improvement.

**Table 3.** Individual results from a 5-fold CV over the CASF-2019 dataset.

Benchmark	RDKit	Amino count	RDKit+amino
Fold 1	0.674/1.444	0.723/1.349	0.781/1.220
Fold 2	0.701/1.407	0.747/1.311	0.797/1.191
Fold 3	0.667/1.448	0.728/1.332	0.775/1.227
Fold 4	0.675/1.470	0.729/1.360	0.774/1.260
Fold 5	0.667/1.474	0.712/1.390	0.768/1.266
Average	0.677/1.449	0.728/1.348	0.779/1.233

a The descriptors are shown separately and in combination (Pearson R/RMSE).

**Figure 1.**
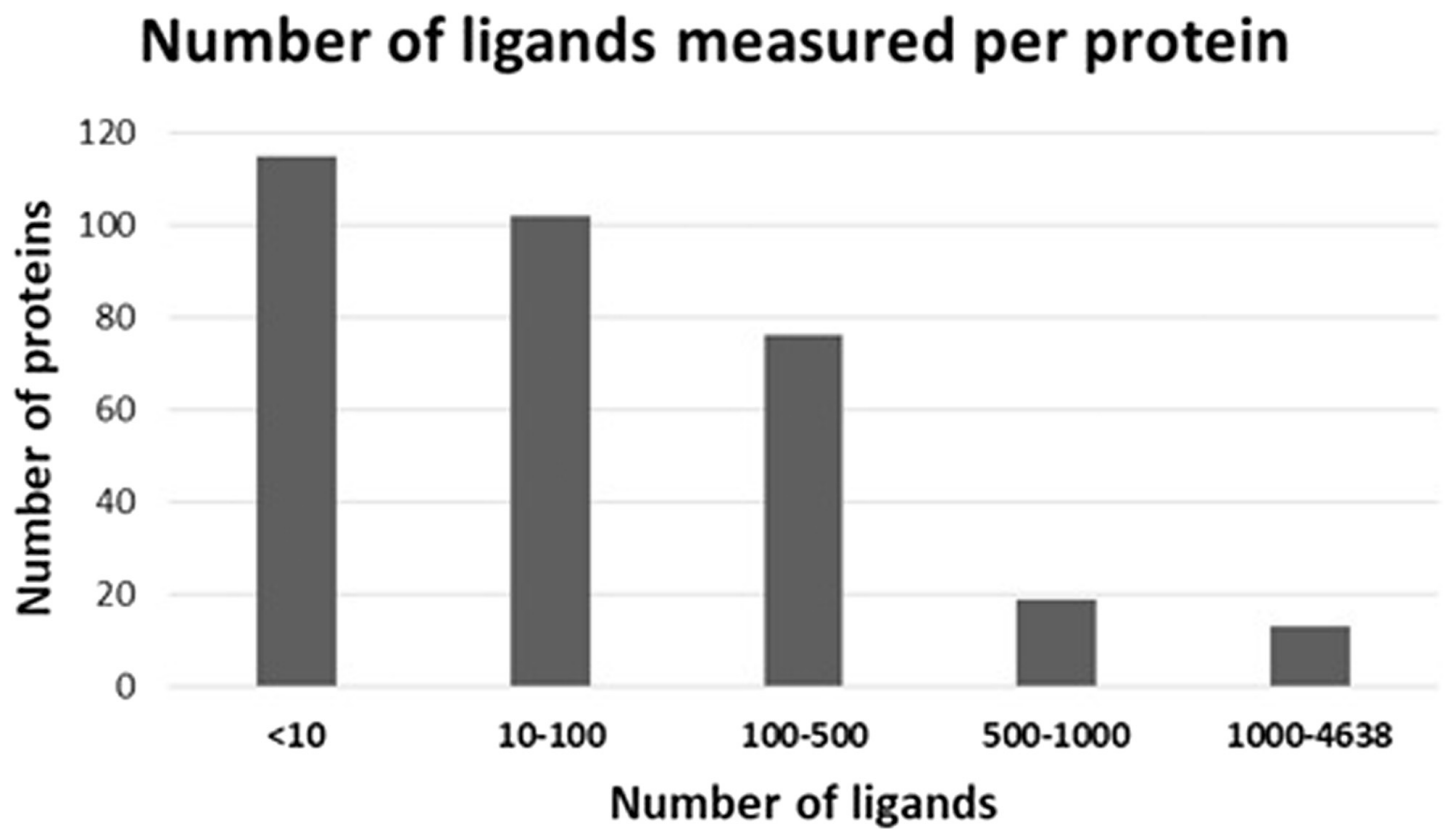
Number of ligands measured per protein in the ChEMBL dataset.

In order to investigate the performance of this approach for predicting affinities for a wider range of ligands, we performed additional experiments. First, we matched protein pockets from the CASF v.2019 dataset with targets in ChEMBL. The available ligands for these ChEMBL targets had their properties calculated using RDKit. Along with the amino acid counts for the pockets, this resulted in a new dataset consisting of 63 281 samples representing unique protein–ligand pairs. Figure 1 illustrates the number of ligands associated with each protein. Since the number of ligands per protein varies significantly, we measure the performance of our descriptors with different thresholds for maximum number of ligands allowed for a single protein (Table 4). Our method appears highly efficient.

**Table 4.** Prediction results for the ChEMBL dataset using amino acid counts based on active sites.

Ligands present	Unique proteins	Samples	RDKit	Amino count (active site)	RDKit+amino
<250	255	10 987	0.663/0.993	0.562/1.096	0.782/0.825
<500	293	24 518	0.679/0.953	0.525/1.105	0.784/0.807
No threshold	325	63 281	0.642/0.961	0.455/1.115	0.775/0.793

a Results presented are the average Pearson R from a 5-fold CV, as well as the RMSE.

The results from the ChEMBL dataset further underscores the usefulness of combining a simple vector representing the amino acid count of the target binding site with ligand properties, when predicting binding affinities. The exact amino acids included in the pockets in this case is not ‘tailored’ to each ligand like they are in the CASF datasets, and they still appear to significantly improve predictions. For many of these proteins, datapoints are limited. For instance, of the 325 proteins, 160 proteins have ≤30 data points available.

Taking the descriptors and the outcomes into account, it is likely that our approach simply makes use of sequence constituent homology rather than estimating a putative binding energy based on amino acids involved in the binding site, and the corresponding potency of ligands based on their descriptors. To investigate this, the experiments using the ChEMBL dataset, which relies on the amino acid count of the active site, were performed again, with the significant difference that the amino acid count was based on the entire protein chain instead of the binding site alone (Table 5).

**Table 5.** Prediction results for the ChEMBL dataset using full protein chain sequence counts.

Ligands present	Unique proteins	Samples	RDKit	Amino count (active site)	RDKit+amino
<250	255	10 987	0.663/0.993	0.562/1.096	0.782/0.827
<500	293	24 518	0.679/0.953	0.525/1.105	0.785/0.806
No threshold	325	63 281	0.642/0.961	0.455/1.115	0.773/0.794

a Results presented are the average Pearson R from a 5-fold CV, as well as the RMSE.

## 4 Discussion

There are many approaches for predicting protein–ligand binding affinity from structural complexes. In this article, we have demonstrated that only a small subset of information along with a tree-based machine learner is required to surpass all but one of the current state-of-the-art approaches in this task using the CASF datasets and a ChEMBL-derived dataset. We used 18 basic ligand features pertaining to structure and chemical properties along with an amino acid count of the polypeptide chain or the active site.

Achieving similar performance using an amino acid count of the full polypeptide chain rather than of the active site alone, makes it likely that protein homology (on an amino acid constituent basis) is leveraged in the learning (cf. Tables 4 and 5). Taken together with the outstanding performance of this simple approach compared with other published methods on the CASF datasets (cf. Tables 1 and 2), we conclude that it is very useful for estimating ligand–protein binding affinity. It is further noteworthy that its utility extends to cases where the spatial relationship between protein and ligand is unknown. However, further investigation is required to delineate the benefits of this approach to specific protein targets.

The fact that our suggested descriptors can be leveraged to such an extent across the CASF datasets does suggest that there is a critical need to design new datasets for the task of predicting protein–ligand complex affinity using machine learning. Such datasets should ensure that predictions are based on correlations between specific interactions between protein and ligand within the binding site. Other studies have also observed the tendency of machine learning models to perform well using nonspatial features when trained on the CASF datasets (Yang *et al.* 2020, Volkov *et al.* 2022). They attribute this fact to bias in the datasets with regards to both proteins and ligands, with one suggested solution to this problem being the construction of larger and less biased datasets.

Future studies employing our proposed approach may adopt more robust datasets derived from ChEMBL and other sources with regards to inclusion criteria of proteins and ligands to reduce any potential bias. However, this study simply set out to demonstrate that the combination of ligand properties and an amino acid count improves affinity prediction for protein–ligand complexes. The attraction of this approach is its simplicity. The predictions presented herein are likely to improve further by the addition of nontrivial information about ligand, protein, or their interaction.

## Supplementary Material

btad502_Supplementary_Data
