# Supplementary material for: Protein–ligand binding affinity prediction exploiting sequence constituent homology
Source: Bioinformatics. 2023 Aug 12;39(8):btad502. doi: 10.1093/bioinformatics/btad502 (PMC10463547; doi:10.1093/bioinformatics/btad502)
Supplement: btad502_Supplementary_Data [file btad502_supplementary_data.pdf]

Protein-Ligand Binding Affinity Prediction Exploiting Homology

Supplementary Materials

RDKit features

Descriptors.TPSA
Descriptors.MolLogP
Descriptors.MolWt
Descriptors.NumValenceElectrons
rdMolDescriptors.CalcNumAmideBonds
Lipinski.HeavyAtomCount
Lipinski.NHOHCount
Lipinski.NOCount
Lipinski.NumHAcceptors
Lipinski.NumHDonors
Lipinski.NumHeteroatoms
Lipinski.NumRotatableBonds
Lipinski.NumAromaticRings
Lipinski.NumSaturatedRings

Lipinski.NumAliphaticRings
Lipinski.NumAromaticHeterocycles
Lipinski.NumAromaticCarbocycles
Lipinski.NumSaturatedHeterocycles

Table S1. Ligand descriptors. Eighteen features were calculated consisting of common structural elements and properties.

PubChem standardiser incompatible files

CASF-2007	'2adm', '1rle', '2j7h', '1a7x', '1mue', '1ksn', '1tyr', '1sl3', '1nw5', '1b8n'
CASF-2013	'1wc1', '3lcv', '3lpp', '3acl', '3l4v', '1nw5', '3l4y', '3l4z', '2h21', '3pgl', '3dx2', '3r24', '3l4x', '3gcp', '2j7h', '3l4u'
CASF-2016	'4zbf', '3lcv', '3l4z', '3gcp', '2j7h', '4qhc', '4qy3', '3l4y', '4bup', '1nw5', '3acl', '2h21', '3l4u', '3r24', '3pgl', '1wc1', '4ymg', '3lpp', '4ufi', '3l4v', '3l4x'
CASF-2019	'4qy3', '3t85', '4zbf', '3ta0', '6d55', '3ta1', '6eqx', '3t70', '6chp', '3tao', '5fyx', '3tay', '6cze', '6bm5', '2h21', '6dh6', '3l4v', '6dj7', '3tb6', '5h5f', '6dh8', '6ced', '6csq', '6dq4', '6cwn', '6css', '3acl', '3t84', '3t82', '6cjv', '3l4u', '5twj', '6d5e', '6d2o', '4ufi', '6d5j', '6czb', '6cfc', '3tcg', '3td4', '4bup', '3pgl', '3lpp', '3t8v', '6dj2', '6d9x', '3l4x', '3gcp', '2j7h', '5bw4', '3r24', '4ymg', '6cn5', '1nw5', '3t83', '1wc1', '6dil', '6dh1', '6d56', '6e4a', '6dif', '6dai', '3l4y', '3l4z', '5kva', '6csr', '6cwh', '6dh7'

Table S2: Files in the refined benchmark datasets incompatible with PubChem's standardiser.
